# Supplementary material for: Protocol for validation of the 4AT, a rapid screening tool for delirium: a multicentre prospective diagnostic test accuracy study
Source: BMJ Open. 2018 Feb 10;8(2):e015572. doi: 10.1136/bmjopen-2016-015572 (PMC5879933; doi:10.1136/bmjopen-2016-015572)
Supplement: Supplementary Appendix 1 [file bmjopen-2016-015572supp001.pdf]

Appendix 1: Detailed consent procedures for incapacity in Scotland and England

Scotland

An appropriate legal proxy (that is, a guardian, welfare attorney, nearest relative, but not a member of the clinical team) will be approached by a member of the clinical team (potentially including researchers who are part of the clinical team) to be asked if they would be willing to consider hearing about a study involving the patient, and to potentially give consent on their behalf. If the proxy assents to hearing more about the study, the study team member responsible for consent will provide the proxy with information about: why they are being approached; the role of a proxy, explanation that acting as a proxy is voluntary; details of the study (as would be given to a participant with capacity). The proxy will be asked for advice on whether the participant should take part in the study and what, in their opinion, the participant's views and feelings would have been on taking part in the project had they retained capacity. Consent forms will be signed when the proxy is physically present. If no appropriate legal proxy can be identified within 96 hours, the patient will not be recruited to the study. This is because in Scotland patients with incapacity cannot be included in studies of nonmedicinal treatments unless there is a guardian, welfare attorney or nearest relative available to give consent.

England

If the patient is incapacitated at study entry then a personal consultee (usually a friend or relative) will be consulted and their opinion sought. The approach used will be similar to that detailed in the previous section when consulting legal proxies in Scotland. If the personal consultee agrees that their friend/relative can enter the study then we would ask them to sign a declaration form. If a personal consultee is not available for consultation then the treating doctor (who will be independent of the research team and of appropriate seniority),

will be asked to act as the nominated consultee and advise on inclusion in the study. If agreement is given it will be recorded on the declaration form.

All Trial Participants (England and Scotland)

All patients who lack mental capacity at the time of enrolment will be approached for consent to remain in the trial at the earliest opportunity once they regain capacity. Research staff have planned contact with study patients on the day of enrolment and only on one further occasion at 12 weeks when they will collect questionnaire data from the patient. If research staff become aware the patient has regained capacity while in hospital then written consent from the patient will be sought at this time. It is likely that in many cases the first contact by the research team will be at 12 weeks either in person or by phone.

The patient will be given the opportunity to either withdraw or remain in the study at this time. If the patient chooses to withdraw from the study they will be given the option of allowing/not allowing the use of data already collected. A patient information sheet will be posted to participants who wish to remain on the study and other patients on request.

If patients have not regained capacity at 12 weeks they will remain in the study based on the advice of the consultee or legal proxy.
